# Ventilatory response to exercise of elite soccer players

**DOI:** 10.1186/2049-6958-9-20

**Published:** 2014-04-02

**Authors:** Adriano Di Paco, Giosuè A Catapano, Guido Vagheggini, Stefano Mazzoleni, Matteo Levi Micheli, Nicolino Ambrosino

**Affiliations:** 1 Pulmonary Rehabilitation and Weaning Center, Auxilium Vitae, Volterra, Pisa, Italy; 2 Rehabilitation Bioengineering Laboratory, ScuolaSuperioreSant’Anna and Auxilium Vitae, Volterra, Italy; 3 Institute of Clinical Physiology, G. Monasterio Foundation/Clinical Research Council Pisa, Pisa, Italy; 4 The BioRobotics Institute, Scuola Superiore Sant’Anna, Pisa, Italy; 5 Physical Education, Sport and Health, University of Florence, Florence, Italy

**Keywords:** Exercise testing, Field performance, Team sports, Training, Ventilatory parameters

## Abstract

**Background:**

The purpose of this study was to evaluate the role of ventilatory parameters in maximal exercise performance in elite soccer players.

**Methods:**

From September 2009 to December 2012, 90 elite soccer players underwent evaluation of lung function test and ergospirometry by means of an incremental symptom-limited treadmill test. Results were analyzed according to i) maximal exercise velocity performed (Hi-M: high-performers, >18.65 km/h; Lo-M: low-performers, <18.65 km/h) and ii) usual role in the team.

**Results:**

Hi-M showed higher peak minute ventilation (

$$\dot{V}E_{\mathrm{peak}}$$:

158.3 ± 19.5 vs 148.0 ± 18.54 L/min, p = 0.0203), and forced expiratory volume at first second (5.28 ± 0.50 vs 4.89 ± 0.52 liters, p < 0.001) than Lo-M, independently of playing role. Moreover, a significant correlation between peak oxygen uptake and

$$\dot{V}E$$

(r = 0.57, p < 0.001) was found.

**Conclusions:**

Ventilatory response plays a role in the assessment of exercise capacity in elite soccer players.

## Background

Playing soccer is the result of combination of technical, tactical, psychological and athletic skills. To assess soccer player performance model, studies have used match analysis systems [1-5], and laboratory and field tests [6-9]. Other studies addressed different roles [10,11], different data tests [12,13], and relationships between endurance field tests and match analysis data [14].

Soccer is a sport characterized by more than 1,000 unpredictable and acyclic changes in activity, each occurring every 3 to 5 seconds, involving up to 40 sprints, tackles and jumps per match [15]. Decelerations, kicking, dribbling, and tackling are additional actions required as well [16]. Therefore, the physical effort imposed on the players is elicited by all these gestures: as a consequence soccer is a highly physiologically demanding sport, with additional stress resulting by frequent matches and high load training sessions are quite high [17-21].

Computerized systems for time-motion analysis have shown that during a match elite soccer players are able to perform 2 to 3 km of high-intensity running (at a speed >15 km/h) and about 0.6 km sprinting (>20 km/h): these distances are 28% and 58% respectively greater than those covered by intermediate level professional players [10]. Technical and tactical skills in soccer are highly dependent on the player physical capacity [22,23].

Relevant effort has been devoted to the evaluation of muscular strength, power, maximal speed, aerobic and anaerobic endurance, but poor attention has been addressed to ventilatory capacity. In most studies cardiorespiratory response to maximal exercise was evaluated using peak oxygen uptake ($\dot{V}O_{2\mathrm{peak}}$) and the corresponding anaerobic threshold value [23-31]. To the best of our knowledge, currently available studies do not focus on ventilatory function as evaluation tool of professional soccer players performance.

In the present study we hypothesized that ventilatory capacity can be a determinant of soccer players exercise capacity. In order to demonstrate our hypothesis the relationship between ventilatory parameters and exercise tolerance was evaluated in a group of elite Italian soccer players using an incremental symptom-limited cardiopulmonary exercise test on a treadmill. Several different measurements reflecting cardiovascular, respiratory, and metabolic response can be recorded during the exercise test. The assessment of ventilatory parameters can contribute to improve training programmes and, consequently, athletic performance.

## Methods

### Subjects

From 2009 to 2012, ninety professional soccer players from five Italian serie A soccer teams were evaluated in the period September-December of each year after completing pre-seasonal training programme in the frame of routine medical evaluations. Each subject provided informed signed consent to the use of their data for scientific purposes.

As expected, no player reported either smoking habit or any relevant disease, with negative chest physical examination. Players were unaware of the aim of the study and researchers performing analysis of results were blind to players’ identity.

### Procedures

#### Lung function test

Dynamic lung volumes were assessed by means of a pneumotachograph (V-Max Encore, Yorba Linda, CA, USA). Predicted values were those of American Thoracic Society [32]. Maximal Voluntary Ventilation (MVV) was estimated multiplying Forced Expiratory Volume at first second (FEV_1_) value by 40 [33].

#### Electrocardiography

Resting and exercise electrocardiography (EKG) was assessed in upright position by means of a 10-lead electrocardiograph (Cardiosoft, GE medical systems, Fairfield, CT, USA) applied on the cardiac screening (six precordial leads) and the posterior wall of the chest (four peripheral leads).

#### Exercise test

An incremental symptom-limited exercise test was performed on a treadmill (Runrace 900, Technogym, Gambettola, Italy) under EKG and pulse oximetry monitoring. Subjects standing on the treadmill breathed through a mask. A continuous “ramp” protocol at constant grade (1%) (starting from 8 km/h, increasing speed by 1 km/h every 60 seconds) was used. The test was stopped when subjects complained of exhaustion. Exercise tolerance was evaluated as the maximal speed reached (Maximal Exercise Velocity: MEV), adjusted according to a modified Kuiper’s equation (Equation 1) [34].

(1) $$\mathrm{MEV}=v_{l}+\left(\frac{n}{6_{0}}\right)\;$$

where *v*_*l*_ represents the speed achieved at the last exercise step and *n* the number of seconds attained during the last stage.

#### Gas measurements

The following variables were measured at peak exercise through breath by breath analysis of inhaled and exhaled gases, by mass flow meter and fast-responding gas analyzer (V-Max Encore, Yorba Linda, CA, USA): Oxygen uptake ($\dot{V}O_{2}$) and $\dot{V}O_{2}$ normalized to body weight, and its relationship with heart rate (HR) (pulse oxygen or $\dot{V}O_{2}/\mathrm{HR}$), CO_2_ production ($\dot{V}CO_{2}$), the physiological dead space to the tidal volume ratio (*V*_*d*_/*V*_*t*_), minute ventilation ($\dot{V}E$), maximal $\dot{V}E$ ($\dot{V}E_{\max}$) expressed as the highest $\dot{V}E$ value recorded either during exercise or at the first time recovery phase, breathing respiratory reserve (BRR% expressed as $\dot{V}E_{\max}$ to MVV ratio). The anaerobic threshold (AT) was estimated by V slope method and ventilatory equivalent method [35]. Anaerobic phase time (APT) was defined as the time spent during $\dot{V}CO_{2}/\dot{V}O_{2}$> 1. Predicted values of HR were computed according to Tanaka et al. [36].

### Statistical analysis

Subjects were categorized into four groups according to their role in the team as reported by the technical staff: forwards (F), central midfielders (CM), central defenders (CD), wide players (WP): goalkeepers were excluded from the study. According to MEV performed in the exercise test, players were divided into two groups (Hi-M: high-performers: able to run at a speed greater than median value of all subjects; Lo-M: low-performers: able to run at a speed lower than that median). Furthermore, the subjects were divided into two groups according to median $\dot{V}E_{\mathrm{peak}}$ (Hi- $\dot{V}E$ and Lo- $\dot{V}E$, respectively).

Linear regression analyses between $\dot{V}E$ and $\dot{V}O_{2}$, HR and $\dot{V}O_{2}$, and HR and MEV, MEV and $\dot{V}O_{2}$ were computed at peak exercise.

Two way analysis of variance (ANOVA) was carried out as follows: 1) dependent variable: $\dot{V}E_{\mathrm{peak}}$; source of variation: role, MEV; 2) dependent variable: FEV_1_; source of variation: role, MEV. All pairwise multiple comparison procedures were carried out using Holm-Sidak method.

Student’s t-test was used for analysing statistical significance of differences in the following parameters: $\;\dot{V}O_{2\mathrm{peak}}$, Body Mass Index (BMI), peak Respiratory Rate (*RR*_*peak*_), peak tidal volume (*Vt*_*peak*_), MEV and $\dot{V}O_{2\mathrm{peak}}/HR_{\mathrm{peak}}$ on Hi-M vs Lo-M and Hi-VE vs Lo-VE, respectively.

Factor analysis was computed on the set of cardiovascular, metabolic, and respiratory variables measured at peak exercise using non-rotated Principal Component Analysis (PCA). PCA is a simple, non-parametric method of extracting relevant information from multivariate datasets. The central idea of PCA is to reduce the dimensionality of a dataset consisting of a large number of interrelated variables, while retaining the variation present in the dataset. This is achieved by transforming into a new variable set, the Principal Components (PCs) which are uncorrelated, and which are ordered so that the first few retain most of the variation present in all the original variables [37].

Multiple linear regression among variables resulting from PCA analysis was carried out. Correlation analysis between variables of interest was computed using Pearson coefficient.

Statistical analysis was carried out using SigmaStat version 3.5 (Systat Software, Inc., USA), except for factor analysis carried out using SPSS v10.1 (SPSS Inc. USA).

## Results

Anthropometric, demographic, and resting physiological characteristics of the whole study population and according to exercise capacity are shown in Table 1. The MEV median value was 18.65 km/h. BMI, height, weight, and age did not show any significant differences between Hi-M and Lo-M. MVV and FEV_1_ were significantly higher in Hi-M than in Lo-M. There was no significant difference in anthropometric and demographic characteristics among different roles played.

**Table 1:** Anthropometric, demographic, and resting physiological characteristics of the whole study population and according to MEV values

	**All**	**Hi-M n = 45**	**Lo-M n = 45**	**p**
**BMI [Kg/m**^**2**^**]**	23.8 ± 1.2	23.6 ± 1.4	24.0 ± 0.9	0.1326
**Height [cm]**	181.8 ± 5.2	184.0 ± 4.9	182.6 ± 5.2	0.059
**Weight [Kg]**	79.4 ± 5.5	80.0 ± 5.6	78.8 ± 5.3	0.3221
**Age [years]**	25.9 ± 4.0	25.2 ± 3.5	26.6 ± 4.4	0.0969
**MVV [L/m]**	201.1 ± 21.7	206.6 ± 20.4	195.6 ± 21.6	0.0155
**FEV**_**1**_**[L]**	5.0 ± 0.5	5.2 ± 0.5	4.9 ± 0.5	0.0152

Values expressed are shown as mean ± SD. Abbreviations as reported in the text.

Table 2 shows the physiological parameters at peak exercise according to exercise capacity. Only ${\dot{V}E}_{\mathrm{peak}}$ was significantly higher in Hi-M than Lo-M, resulting from non significantly greater Vt_peak_ and RR_peak_, and non significantly lower V_d_/V_t_ in Hi-M than Lo-M subjects.

**Table 2:** Physiological parameters at peak exercise of subjects according to MEV

	**All**	**Hi-M n = 45**	**Lo-M n = 45**	**p**
**MEV [km/h]**	18.5 ± 1.1	19.4 ± 0.6	17.6 ± 0.7	0.0000
$\dot{V}E_{\mathrm{peak}}\left[L/\min\right]$	151.7 ± 19.4	156.2 ± 19.8	147.1 ± 17.9	0.0249
**(V**_**d**_**/V**_**t**_**)**_**peak**_	0.10 ± 0.03	0.09 ± 0.04	0.11 ± 0.03	0.0548
**RR**_**peak**_**[breaths/min]**	53.3 ± 4.9	54.5 ± 5.1	52.6 ± 4.6	0.0773
**Vt**_**peak**_**[L/min]**	2.87 ± 0.45	2.92 ± 0.42	2.84 ± 0.46	0.3848
**HR**_**peak**_**[b/min]**	186.0 ± 9.5	186.2 ± 9.6	185.7 ± 9.4	0.7911
$\dot{V}O_{2\mathrm{peak}}\left[\mathrm{mL}/\min/\mathrm{Kg}\right]$	63.3 ± 5.3	63.3 ± 5.3	63.2 ± 5.4	0.9359
**BRR[L]**	41.9 ± 20.8	42.8 ± 20.1	41.0 ± 21.4	0.6726
**BRR%**	79.0 ± 9.3	79.1 ± 9.5	78.8 ± 9.1	0.8750

Values are shown as mean ± SD. Abbreviations as reported in the text.

During the test, all players reached 97.9 ± 4.7% of their predicted maximal HR without any significant difference between groups.

Table 3 shows resting and at peak exercise physiological characteristics according to $\dot{V}E_{\mathrm{peak}}$. Median $\dot{V}E_{\mathrm{peak}}$ was 153.05 L/min. Hi-VE showed significantly higher ${\dot{V}O}_{2\mathrm{peak}}$, $\dot{V}O_{2\mathrm{peak}}/{\mathrm{HR}}_{\mathrm{peak}}$, MEV, BRR%, Vt_peak_ and FEV_1_ than Lo-VE subjects.

**Table 3:** **Resting and at peak exercise physiological characteristics of subjects according to**$\dot{V}E_{\mathrm{peak}}\mathrm{values}$

	**Hi-VE n = 45**	**Lo-VE n = 45**	**p**
**MEV [km/h]**	18.9 ± 0.9	18.1 ± 1.2	0.0008
$\dot{V}E_{\mathrm{peak}}\left[L/\min\right]$	166.3 ± 10.4	137.1 ± 14.8	0.0000
**(V**_**d**_**/V**_**t**_**)**_**peak**_	0.09 ± 0.03	0.10 ± 0.03	0.0741
**RR**_**peak**_**[breaths/min]**	53.8 ± 5.1	52.7 ± 4.6	0.2617
**Vt**_**peak**_**[L/min]**	3.11 ± 0.31	2.62 ± 0.34	0.0000
**HR**_**peak**_**[b/min]**	185.8 ± 8.9	186.1 ± 10.0	0.8946
$\dot{V}O_{2\mathrm{peak}}/HR_{\mathrm{peak}}\left[\mathrm{mL}/\min/\mathrm{bb}\right]$	28.3 ± 4.0	25.4 ± 3.8	0.0004
$\dot{V}O_{2\mathrm{peak}}\left[\mathrm{mL}/\min/\mathrm{Kg}\right]$	65.7 ± 4.4	60.8 ± 5.3	0.0000
**BRR[L]**	34.5 ± 18.0	49.3 ± 20.8	0.0006
**BRR%**	83.4 ± 7.8	74.6 ± 8.7	0.0000
$RR_{\mathrm{peak}}\left[\dot{V}CO_{2}/\dot{V}O_{2}\right]$	1.08 ± 0.17	1.10 ± 0.10	0.4576
**MVV[L/min]**	208.0 ± 18.0	194.2 ± 22.9	0.0020
**FEV**_**1**_**[L]**	5.2 ± 0.4	4.8 ± 0.6	0.0021

**Figure 1:**
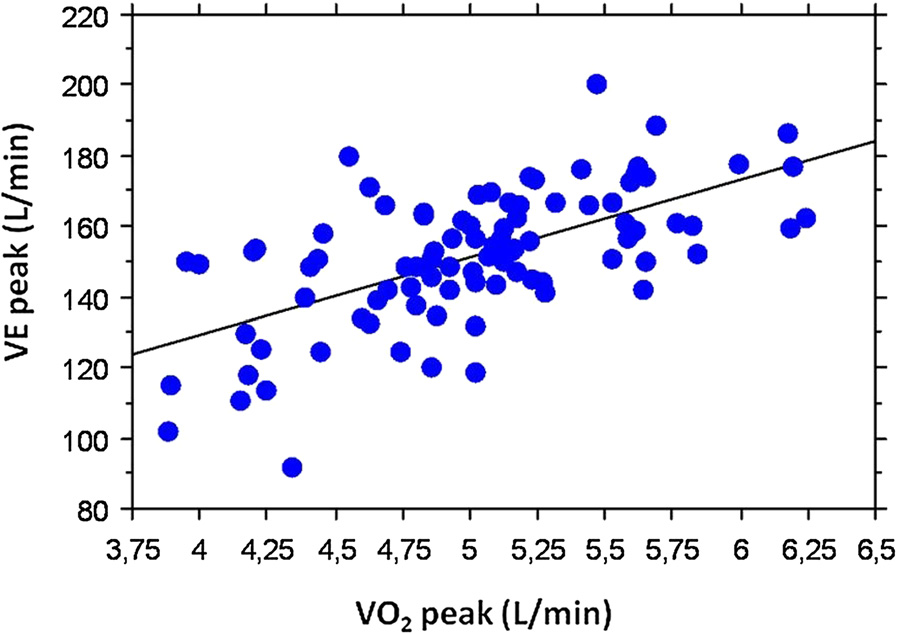
**Relationship between minute ventilation and oxygen uptake at peak exercise as shown by the equation:*VE***_***peak***_ = 41.76 + (21.88 * *VO*_**2*peak***_**) ** ***r* = 0.619,** ***p* < 0.001.**

Values are shown as mean ± SD. Abbreviations as reported in the text.

$$\dot{V}E_{\mathrm{peak}}$$

was significantly correlated to $\dot{V}O_{2\mathrm{peak}}$ (r = 0.619, p = 0.001) (Figure 1) and MEV was significantly correlated to $\dot{V}O_{2\mathrm{peak}}$ (r = 0.267, p = 0.011). No statistical significant correlation was found between *HR*_*peak*_ and MEV.

A weak, although significant correlation between *V*_*d*_/*V*_*t*_ and $\dot{V}E$ at peak exercise was found in all players (r = −0.282; p = 0.007). No significant differences in $\dot{V}O_{2\mathrm{peak}}$, MEV and BRR were found among different roles, whereas mean MVV was significantly lower in F than in CM (193.3 ± 21.2 vs 206.1 ± 19.9 liters respectively, p = 0.043). ANOVA showed that the difference in the mean value of ${\dot{V}E}_{\mathrm{peak}}$ and FEV_1_ among the different levels of role of players was not significantly different after allowing for the effects of differences in MEV (p = 0.377 and p = 0.543, respectively).

Factor analysis using the PCA on the variables measured at peak exercise and at rest was carried out. Table 4 reports the distribution along the first three components of the following variables: $\dot{V}E_{\mathrm{peak}}$, $\dot{V}O_{2\mathrm{peak}}$, (*V*_*d*_/*V*_*t*_)_peak_, *HR*_*peak*_, BRR_peak_, RR_peak_, $\dot{V}O_{2\mathrm{peak}}/HR_{\mathrm{peak}}$, FEV_1_. The first three components accounted for 74.33% of cumulative variance. Multiple linear regression on most representative variables on each component deriving from PCA at peak exercise is represented by the following equation:

(2) $$\begin{array}{l} VO_{2\mathrm{peak}}=1.748+\left(0.012\times VE_{\mathrm{peak}}\right)\\ \;+\left(0.299\times \mathrm{FE}V_{1}\right)-\left(0.003\times \mathrm{BRR}\right)\;R\\ \;=0.641\;p<0.05\; \end{array}$$

**Table 4:** PCA component matrix (peak values)

	**Component**		
	**1**	**2**	**3**
$\dot{V}O_{2\mathrm{peak}}$	0.950	0.014	0.081
$\dot{V}O_{2\mathrm{peak}}$	0.859	−0.217	−0.088
$\dot{V}O_{2\mathrm{peak}}/HR_{\mathrm{peak}}$	0.853	−0.182	0.305
$\dot{V}E_{\mathrm{peak}}$	0.720	0.147	−0.518
**RR**_**peak**_	−0.305	0.362	−0.665
**HR**_**peak**_	−0.180	0.406	−0.304
**BRR**	−0.197	0.675	0.688
**(V**_**d**_**/V**_**t**_**)**_**peak**_	−0.341	−0.545	0.301
**FEV**_**1**_	0.432	0.817	0.214

Equation (2) represents a statistically significant relationship between *VO*_2*peak*_ and *VE*_*peak*_, *FEV*_1_ and *BRR*.

## Discussion

The novelty of this study is represented by the analysis of ventilatory parameters as evaluation tool of professional soccer players performance. Our elite soccer players were evaluated during seasonal activity. The main finding of our study is that ${\dot{V}E}_{\mathrm{peak}}$ and FEV_1_ but not ${\dot{V}O}_{2\mathrm{peak}}$ are the main determinants for discriminating high and low performers: nevertheless, neither Hi-M nor Lo-M were ventilatory limited. Therefore, our results are in agreement with available literature about elite athletes: the main limitation to maximal performance is represented by cardiovascular limit. Furthermore, our data highlight the key role of resting dynamic ventilatory parameters like FEV_1_ as predictive factor of maximal exercise capacity, indicating the need to include lung function test in the routine evaluation of these elite soccer players.

In our study population measured $\dot{V}O_{2\mathrm{peak}}$ is in agreement with average values reported in previous studies on international elite soccer players [11]. Currently, ventilatory parameters are poorly investigated in these athletes and our study provides a contribution to this field.

Significant relationships between $\dot{V}E_{\mathrm{peak}}$, FEV_1_ and BRR with exercise capacity were found, and these parameters were significantly different between high and low performers. This finding offers a new insight in this field as it provides a quantitative relationship between dynamic ventilatory parameters and exercise performance in these athletes. Indeed, $\dot{V}O_{2\mathrm{peak}}$ described by a linear combination of most representative ventilatory parameters resulting by PCA analysis (i.e., $\dot{V}E_{\mathrm{peak}}$, FEV_1_ and BRR) corresponds to a physiological relationship among these variables. Therefore, the physiological parameters obtained from a simple test like spirometry might be used to distinguish high from low performers.

The significant correlation found between (*V*_*d*_/*V*_*t*_)_peak_ and $\dot{V}E_{\mathrm{peak}}$ on the whole study population represents an interesting finding: high values of $\dot{V}E_{\mathrm{peak}}$ found in Hi-M elite soccer athletes correspond to low values of (*V*_*d*_/*V*_*t*_)_peak_. High ventilatory efficiency is more important in achieving better performance than VE. These parameters depend also on anthropometric and demographic characteristics which in our study were not significantly different between groups of players, independently of exercise capacity, ventilatory capacity, or role played.

Our study did not find any significant difference in physiological parameters among different playing roles. This may be related to lack of specific training among the roles.

### Limitation of the study

The subjective reason to stop exercise, either dyspnoea, muscular fatigue or both, was not recorded, despite most subjects informally reported muscular fatigue. This is not surprising given the lack of ventilatory limitation to exercise shown by these athletes.

## Conclusions

Respiratory parameters can play a determinant role in qualitative and quantitative evaluation of professional soccer players performance as it was confirmed by results discussed so far. Further studies should be addressed to investigate the impact of different training programmes on dynamic ventilatory parameters.

## Competing interests

The authors declare that they have no competing interests.

## Authors’ contributions

ADP conceived the study, participated in its design, collected data and helped to draft the manuscript. SM participated in the design of the study, performed the statistical analysis and helped to draft the manuscript. GAC and MLM participated in the design of the study. GV participated in the design of the study and performed the statistical analysis. NA coordinated the study and helped to draft the manuscript. All authors read and approved the final manuscript.
